# The *pvc* Operon Regulates the Expression of the *Pseudomonas aeruginosa* Fimbrial Chaperone/Usher Pathway (*Cup*) Genes

**DOI:** 10.1371/journal.pone.0062735

**Published:** 2013-04-30

**Authors:** Uzma Qaisar, Liming Luo, Cecily L. Haley, Sean F. Brady, Nancy L. Carty, Jane A. Colmer-Hamood, Abdul N. Hamood

**Affiliations:** 1 Department of Immunology and Molecular Microbiology, School of Medicine, Texas Tech University Health Sciences Center, Lubbock, Texas, United States of America; 2 Laboratory of Genetically Encoded Small Molecules, The Rockefeller University and Howard Hughes Medical Institute, New York, New York, United States of America; The Scripps Research Institute and Sorrento Therapeutics, Inc., United States of America

## Abstract

The *Pseudomonas aeruginosa* fimbrial structures encoded by the *cup* gene clusters (*cupB* and *cupC*) contribute to its attachment to abiotic surfaces and biofilm formation. The *P. aeruginosa pvcABCD* gene cluster encodes enzymes that synthesize a novel isonitrile functionalized cumarin, paerucumarin. Paerucumarin has already been characterized chemically, but this is the first report elucidating its role in bacterial biology. We examined the relationship between the *pvc* operon and the *cup* gene clusters in the *P. aeruginosa* strain MPAO1. Mutations within the *pvc* genes compromised biofilm development and significantly reduced the expression of *cupB1-6* and *cupC1-3*, as well as different genes of the *cupB*/*cupC* two-component regulatory systems, *roc1/roc2*. Adjacent to *pvc* is the transcriptional regulator *ptxR*. A *ptxR* mutation in MPAO1 significantly reduced the expression of the *pvc* genes, the *cupB/cupC* genes, and the *roc1/roc2* genes. Overexpression of the intact chromosomally-encoded *pvc* operon by a *ptxR* plasmid significantly enhanced *cupB2*, *cupC2*, *rocS1*, and *rocS2* expression and biofilm development. Exogenously added paerucumarin significantly increased the expression of *cupB2*, *cupC2*, *rocS1* and *rocS2* in the *pvcA* mutant. Our results suggest that *pvc* influences *P. aeruginosa* biofilm development through the *cup* gene clusters in a pathway that involves paerucumarin, PtxR, and different *cup* regulators.

## Introduction

*Pseudomonas aeruginosa* is a versatile gram-negative opportunistic pathogen that causes severe acute and chronic infections at different sites within the body including; urinary tract, skin (burn or surgical wounds), and the respiratory tract [1]–[3]. Burn patients, individuals with cystic fibrosis (CF), patients in intensive care units, and intubated patients (mechanical ventilator) are susceptible to *P. aeruginosa* infection [1], [4], [5]. *P. aeruginosa* also causes serious infection in immunocompromised patients including cancer patients undergoing chemotherapy and HIV infected patients [2], [4]. Damage caused by *P. aeruginosa* is due to the production of numerous cell-associated and extracellular factors [3], [5], [6]. Extracellular (secreted) virulence factors include exotoxin A, elastases (LasB and LasA), alkaline protease, type III secretion system effector molecules, and pyocyanin; while cell-associated virulence factors include the flagellum, type IV pili, exopolysaccharide (EPS), and lipopolysaccharide [3], [5], [6]. At different infection sites, *P. aeruginosa* exist within biofilms – sessile, complex and highly structured communities that are surrounded by EPS matrix [7]–[9]. Within the biofilm, the bacteria are protected from the effect of the host immune response [7]–[9]. Additionally, bacterial resistance to different antibiotics is increased considerably within the biofilm [10]. Different *P. aeruginosa* infections including endocarditis, otitis media, chronic pneumonia in CF patients, and chronic wound infections involve biofilm development [11]–[15]. *P. aeruginosa* biofilms also develop on different medical devices such as central venous catheters, intrauterine devices, mechanical heart valves, contact lenses, and indwelling urinary catheters [7], [13], [16]–[18].

Biofilm development by *P. aeruginosa* occurs in several stages [19]–[21]. In the initial stages, bacteria attach reversibly to abiotic or biotic surfaces, followed by irreversible attachment and maturation of the biofilm [19]–[21]. During the maturation stage, the bacteria multiply, form microcolonies, and produce the EPS matrix around the microcolonies [19]–[21]. The final stage involves dispersion and focal dissolution of the biofilm [19]–[21]. Attachment is accomplished through two cell-associated structures, the flagella and type IV pili [21]–[23]. Type IV pili also allow the bacteria to climb a biofilm formed by other bacteria and colonize the top of that biofilm [22]. Biofilm formation by *P. aeruginosa* also involves fimbrial structures that are components of and assembled on the outer surface of the bacteria by the conserved chaperone/usher pathways termed Cup [21], [24], [25]. The Cup consist of an usher (outer membrane protein), one or two chaperone (periplasmic protein), and the fimbrial subunits [25], [26]. Previous studies identified four *P. aeruginosa cup* gene clusters, *cupA1-5, cupB1-6, cupC1-3*, and *cupE1-6,* that code for an usher, one or two chaperones, and at least one fimbrial subunit [26]–[28]. Within these clusters, *cupA1* and *cupA4*, *cupB1* and *cupB6*, and *cupC1* code for the major fimbrial subunits; *cupA2* and *cupA5*, *cupB2* and *cupB4*, and *cupC2* encode the chaperones; and *cupA3*, *cupB3*, and *cupC3* code for the usher proteins [26], [27]. While *cupE4* and *cupE5* encode a chaperon and usher, respectively; *cupE1*, *cupE2*, *cupE3*, and *cupE6* encode proteins that have none of the characteristics of other archetypal systems of the chaperon usher pathway [28]. An additional cluster, *cupD1-5*, exists in the *P. aeruginosa* strain PA14 [29]. In this cluster, *cupD1* codes for the major fimbrial subunit, *cupD2* codes for the chaperon, and *cupD3* codes for an outer membrane usher [29]. *cupD4* and *cupD5* code for a predicted adhesin and a secondary chaperon, respectively [29]. Further studies described the two *roc* regulatory systems that control the expression of *cupB* and *cupC* genes [30], [31].

Pyoverdine is a high affinity iron-chelating peptide derived molecule that functions as the primary siderophore in *P. aeruginosa* [32]–[34]. The pyoverdine molecule consists of a variable cyclic peptide moiety and a conserved bicyclic chromophore [32]–[34]. Several *P. aeruginosa* genes encode enzymes required for the synthesis of the pyoverdine molecule [34]. Besides pyoverdine, Stintzi *et al.* [35], identified pseudoverdine; a fluorescent bicyclic metabolite that structurally resembles the pyoverdine chromophore. Enzymes synthesizing the pseudoverdine are encoded by the *pvcA-D* gene cluster [36]. However, further studies indicated that the *pvcA-D* gene cluster encode enzymes involved in the synthesis of a novel secondary metabolite termed paerucumarin [37], rather than pyoverdine. So far, no physiological role has been assigned for either the *pvcA-D* gene cluster or paerucumarin [36]. The *pvcA-D* gene cluster is located downstream of *ptxR*, which encodes a transcriptional activator that regulates the expression of different *P. aeruginosa* genes including *toxA* and the quorum sensing (QS) genes [36], [38], [39]. *pvcA-D* expression is negatively regulated by iron and positively regulated by PtxR [36].

In this study, we examined the effect of the *pvcA-D* gene cluster on biofilm development in the *P. aeruginosa* strain MPAO1. Our results confirmed that *pvcA-D* constitutes the *pvc* operon, and that *pvc* influences biofilm development by regulating the expression of *cupB* and *cupC*. Additionally, the *pvc* operon mediates its effect on the *cupB* and *cupC* genes through the secondary metabolite, paerucumarin in a pathway that includes *ptxR*.

## Materials and Methods

### Strains, Plasmids and General Growth Conditions

*Pseudomonas aeruginosa* strains and plasmids used in this study are listed in Table 1. Bacterial cultures were grown in Luria Bertani (LB) broth at 37°C with shaking at 250 r.p.m. for 14–16 h. These cultures were used to inoculate fresh LB broth or tryptone broth (1% tryptone, 5 mM MgCl_2_, 2.5 mM KCl_2_ and 25 µM FeCl_3_). Plasmids were isolated using FastPlasmid Mini Kit (5 Prime) and electroporated into *P. aeruginosa* strains using a Gene Pulser (Bio-Rad Laboratories) as previously described [40]. Antibiotics were added to the media at the following concentrations: 300 µg carbenicillin ml^−1^ and 60 µg tetracycline ml^−1^.

**Table 1:** Bacterial strains and plasmids used in this study.

Strain or Plasmid	Description	Reference/Source
*Strains*		
MPAO1	*P. aeruginosa* prototrophic laboratory strain	[66], [67], UWGC
PW4830	*pvcA*-F05::IS*lacZ*/hah; out of frame fusion in MPAO1; Tet^r^	[67], UWGC
PW4832	*pvcB*-G05::IS*lacZ*/hah; out of frame fusion in MPAO1; Tet^r^	[67], UWGC
PW4833	*ptxR-*P08::IS*lacZ/*hah; out of frame fusion in MPAO1; Tet^r^	[67], UWGC
PW6105	*rocS1-*P09:IS*lacZ/*hah; out of frame fusion in MPAO1; Tet^r^	[67], UWGC
PW7672	*rocS2-*P07::IS*lacZ/*hah; out of frame fusion in MPAO1; Tet^r^	[67], UWGC
MPAO1Δ*pvcCD*	MPAO1 carrying a 600-bp *BglII* deletion encompassing portions of *pvcC* and *pvcD*; Tet^r^	This study
*Plasmids*		
pCR®2.1-TOPO®	General cloning vector; Cb^r^, Km^r^	Invitrogen
pCR2.1-1.8	pCR®2.1-TOPO® carrying the 1.8-kbp PstI *P. aeruginosa* stability fragment; Cb^r^, Km^r^	This study
pLL1	pCR2.1-1.8 carrying intact *pvcA* on a 1730-bp fragment from MPAO1; Cb^r^, Km^r^	This study
pLL2	pCR2.1-1.8 carrying intact *pvcB* on a 1200-bp fragment from MPAO1; Cb^r^, Km^r^	This study
pLL4	pCR2.1-1.8 carrying intact *rocS1* on a 4060-bp fragment from MPAO1; Cb^r^, Km^r^	This study
pLL5	pCR2.1-1.8 carrying intact *pvcAB* on a 2930-bp fragment from MPAO1; Cb^r^, Km^r^	This study
p18.230	pKT230 carrying pUC18; Cb^r^, Km^r^	[39]
pJAC7-1	pUC19 containing *ptxR* on a 2.1-kbp *KpnI-BglII* fragment carried in pKT230; Cb^r^, Km^r^	[39]
pMRP9-1	pUCP18 carrying a gene encoding enhanced green fluorescent protein (GFP); Cb^r^	[43]
pAH54	pUC18 carrying a 5.4 kb *HindIII-EcoRI* fragment carrying *ptxR, pvcD, pvcC,* and part of *pvcB*	[39]
pNC80	pAH54 with a Tet cassette replacing the 600 bp *BglII* fragment that carries the entire *pvcD*and part of *pvcC*; Tet^r^	This study

Cb, carbenicillin;

Km, kanamycin;

r , resistant;

Tet, tetracycline.

### Biofilm Development and Analysis

We used the air-liquid interface method of biofilm development described by Kulasekara *et al.* [30] with some modification. Overnight cultures of *P. aeruginosa* strains were subcultured at a starting OD_600_ of 0.02 into 3 ml of tryptone broth in polypropylene tubes (BD Biosciences) and incubated under static condition at 37°C for 16 h. Biofilms developed at the air-liquid interface were washed with sterile distilled water to remove planktonic cells. Four ml of 1X PBS was added to each tube and vigorously vortexed to release the cells from the biofilm. The bacterial suspensions were serially diluted tenfold in 1X PBS. A 10-µl aliquot of each dilution was spotted on LB agar plates in triplicates and the agar plates were incubated at 37°C for 16 h. The number of microorganisms (colony forming units, CFU) was calculated using the formula: CFU × dilution factor × 100. Each experiment was repeated three times.

To visualize the biofilms, plasmid pMRP9-1 (Table 1), which expresses GFP was introduced into MPAO1, PW4830 and PW4832, the transposon mutants of *pvcA* and *pvcB* (Table 1), by electroporation. Prior to visualization, the biofilms within the polypropylene tubes were washed with distilled water. Biofilms were examined by CLSM using an Olympus 1X71 Fluoview 300 confocal laser scanning microscope (Olympus). Image stacks were analyzed with the COMSTAT program using MATLAB [41] and various aspects of biofilm structure were determined.

### Plasmid Construction

The 1.8-kbp *PstI* fragment that allows *E. coli* plasmids to stably replicate in *P. aeruginosa* was cloned into the cloning vector pCR2.1®-TOPO® (Invitrogen) generating pCR2.1-1.8. For complementation experiments, DNA fragments containing intact *pvcA* (1730 bp), *pvcB* (1200 bp), *pvcAB* (2930 bp), and *rocS1* (4060 bp) were synthesized from the MPAO1 chromosome by PCR using specific primers (Table S1) and cloned individually into pCR2.1-1.8. In the resulting recombinant plasmids (pLL1, pLL2, pLL5, and pLL4, respectively), the genes are constitutively expressed in *P. aeruginosa* from the *lac* promoter. Construction of the recombinant plasmids was confirmed by restriction digestion and DNA sequence analysis. Recombinant plasmids were introduced into *P. aeruginosa* strains by electroporation.

### Construction of the *pvcC-D* Isogenic Mutant

MPAO1Δ*pvcCD* was constructed from MPAO1 by the gene replacement technique as previously described [39]. A 600-bp *BglII* fragment that carries the entire *pvcC* gene and part of *pvcD* was deleted from pAH54 and replaced by a 1.4 kb fragment that carries the tetracycline resistance gene [39]. The recombinant plasmid pNC80 was introduced into PAO1 by electroporation and the transformants were screened as previously described [39], [40]. Construction of the mutant was confirmed by PCR and Southern blot hybridization.

### Reverse Transcription Quantitative PCR (RT-qPCR)

Overnight cultures of different *P. aeruginosa* strains were subcultured in fresh LB broth to an OD_600_ of 0.02 and incubated at 37°C with shaking for 16 h. Cultures were then mixed with twice the volume of the culture of RNAprotect Bacteria Reagent (QIAGEN) for 5 min at room temperature. The cells were pelleted and stored at −80°C. Bacterial pellets were first lysed with lysozyme and proteinase K for 15 min at room temperature and the RNA was subsequently extracted using the RNeasy Mini Kit (QIAGEN) according to the manufacturer’s recommendations. The RNA solution was then digested with the RNase-free DNase Set (QIAGEN). RNA was purified from DNase by the RNA cleanup protocol (QIAGEN) with the exception that on-column DNase digestion was applied to eliminate any remaining traces of genomic DNA. RNA was quantified by NanoDrop® spectrophotometer (NanoDrop Products) and the integrity of the RNA was assessed using RNA Nano Chip on an Agilent 2100 Bioanalyzer (Agilent).

Synthesis of cDNA from the extracted RNA was performed using the QuantiTect Reverse Transcription Kit (QIAGEN). A 200-ng aliquot of cDNA was mixed with SYBR Green PCR Master Mix (Life Technologies) and 250 nM of specific primer (Table S1). Amplification and detection of the product was conducted using StepOne Plus real-time PCR system (Life Technologies). For each experiment, we used three independent biological replicates for RNA extraction. Additionally, each PCR reaction was set up in triplicate. The quantity of cDNA in different samples was normalized using 30S ribosomal RNA (*rplS*) as an internal standard. Gene expression analysis was performed using StepOne Plus software version 2.2.2 (Life Technologies). Positive control samples containing genomic DNA as a template and negative control samples containing RNA as a template were included in the experiment (data not shown).

### Thin Layer Chromatography (TLC)

Overnight cultures of *P. aeruginosa* strains were subcultured in fresh LB broth to an OD_600_ of 0.02 and incubated at 37°C for 16 h with shaking. Cells were pelleted and supernatant fractions were separated. A 400 µl sample of each supernatant was added to 1200 µl of acidified ethyl acetate and vortexed for 2 min. Samples were centrifuged at 10,600×*g* for 3 min to separate phases. The upper phase was collected, vacuum dried and dissolved in 30 µl of 1∶1 ethyl acetate:acetonitrile. Eight µl of each sample was loaded onto an activated high performance TLC gel 60F_254_ (EMD). TLC plates were developed in a mixture of methylene chloride, acetonitrile and 1,4-dioxane (17∶2:1) for 2 h. Plates were illuminated with long wave UV light and photographed with a FluorChem 8000 imaging system (Alpha Innotech).

### Purification of Paerucumarin and Synthesis of Pseudoverdine

Paerucumarin was purified from the supernatant of PAO1 as previously described in detail by Clarke-Pearson & Brady [37]. Pseudoverdine was synthesized through a commercial source (Eburon Organics, Lubbock, TX) based on the previously reported structure of the molecule [35], [37].

## Results

### *pvcA* Enhances Biofilm Formation by MPAO1

**Figure 1:**
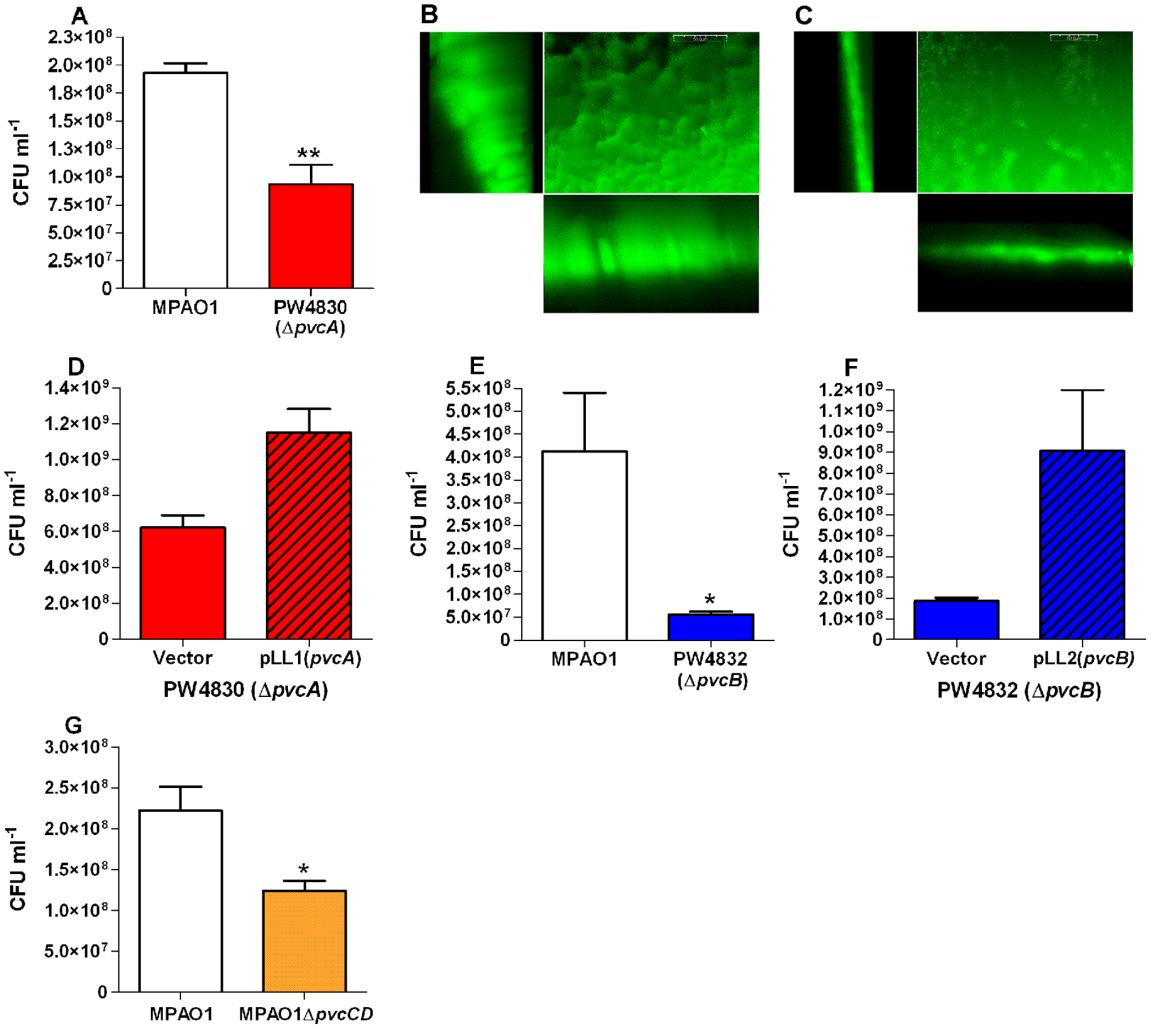
*pvc* genes affect biofilm development in MPAO1. **A.** Mutation in *pvcA* reduces biofilm development in the MPAO1 isogenic mutant PW4830. Strains were transformed with pMRP9-1, which expresses GFP. Overnight cultures were subcultured into tryptone broth as described in Materials and Methods. Bacterial biofilms formed in a ring at the air-liquid interface were washed to remove planktonic cells and the biofilm cells were removed by vortexing in PBS, diluted tenfold, and plated to quantify the viable microorganisms within the biomass (CFU ml^−1^). **B and C.** Representative photomicrographs of the biofilms formed by (**B**) MPAO1 and (**C**) PW4830 (Δ*pvcA*) visualized with CLSM at 40X magnification. Z slices of 0.5 µm were generated; the *zy* and *zx* planes of the Z images are shown to the left and below the flat field, respectively. Bars equal 50 nm. **D–G.** Biofilms were developed and CFU assayed as described in A. **D.** Plasmid pLL1 carrying intact *pvcA* constitutively expressed from the *lac* promoter complements the defect of PW4830 in biofilm formation. Strains were transformed with pLL1 or pCR2.1-1.8 (vector control). **E.** Mutation in *pvcB* also reduces biofilm development in the MPAO1 isogenic mutant PW4832. **F.** Plasmid pLL2 carrying intact *pvcB* constitutively expressed from the *lac* promoter complements the defect of PW4832 (Δ*pvcB*) in biofilm formation. Strains were transformed with pLL1 or pCR2.1-1.8 (vector control). **G.** Mutation in *pvcC-D* reduces biofilm development in the MPAO1 isogenic mutant MPAO1Δ*pvcC-D*. Values in A and D-G represent the average of three independent experiments ± standard error of the mean (SEM). Statistical significance in viable biomass between the strains was calculated by Student’s unpaired *t-*test. *P*<0.05 (*); *P*<0.01 (**).

*P. aeruginosa* forms biofilms at different infection sites including acute and chronic wounds and the lungs of cystic fibrosis patients [11]–[15], [42]. Biofilm formation, which is a major virulence attribute in *P. aeruginosa*, is controlled by numerous characterized genes and possibly additional, as yet uncharacterized, genes. To determine if *pvcA* influences biofilm formation, we utilized the previously described static biofilm system in which *P. aeruginosa* forms a ring of biofilm at air-liquid interface [24], [26]. While MPAO1 and PW4830 (Δ*pvcA*; Table 1) formed biofilm rings, the amount of the biofilm, as measured by CFU ml^−1^, formed by PW4830 was significantly less than that formed by MPAO1 (Figure 1A). We obtained similar results when we analyzed the biofilms using the staining assay (Text S1, Figure S1). To analyze the structure of the biofilm, we introduced pMRP9-1 (Table 1), which carries the gene for green fluorescent protein (GFP) [43], into both strains. Again, while both strains formed biofilm rings, the amount of biofilm formed by PW4830/pMRP9-1 was significantly less than that formed by MPAO1/pMRP9-1 (data not shown). Visualization of the biofilms by confocal laser scanning microscopy (CLSM) revealed that MPAO1/pMRP9-1 formed a dense, well-developed biofilm with clusters of pillar-like microcolonies that extended to a considerable height (Figure 1B). In contrast, PW4830/pMPR9-1 formed a flat, less developed biofilm with sparse microcolonies (Figure 1C). Quantitative analysis of the biofilms using the COMSTAT program [41] revealed significant differences between them in the mean thickness and total biovolume. The total biovolume of MPAO1/pMRP9-1 biofilm was 2.3 µm^3^/µm^2^ while that of PW4830/pMRP9-1 was 0.079 µm^3^/µm^2^ (Table 2). Similarly, the mean thickness of MPAO1/pMRP9-1 biofilm was 9.946 µm while that of PW4830/pMRP9-1 was only 0.049 µm (Table 2).

**Table 2:** Quantification of the effect of *pvcA* mutation on biofilm formation.

Variable^a^	Image stacks (#)^b^	Total biovolume (µm^3^/µm^2^)^c^	Mean thickness (µm)^d^	Roughness coefficient^e^	Total surface area×10^7^ (µm^2^)^f^	Surface to volume ratio (µm^2^/µm^3^)^g^
MPAO1	50	2.300±0.25	9.946±0.98	1.625±0.05	0.476±0.06	1.920±0.13
PW4830	50	0.079±0.00	0.049±0.00	1.988±0.00	0.023±0.00	2.697±0.05
PW4830 vs. MPAO1		*P* = 0.0009	*P* = 0.0005	*P* = 0.0027	*P* = 0.0020	*P* = 0.0053

a Strains, both carrying pMRP9-1, were grown for 16 h at 37°C without shaking.

b Image stacks were acquired in triplicate at 10X magnification and analyzed using the COMSTAT program; values represent the mean ± SEM.

c Biomass of the biofilm.

d Spatial size of the biofilm.

e Variation in the thickness of the biofilm, or heterogeneity.

f Total of the area occupied in each image stack.

g Portion of the biofilm exposed to nutrients; biovolume divided by the surface area of the substratum.

To confirm these findings, we conducted complementation experiments. A 1730-bp DNA fragment containing intact *pvcA* was synthesized from the MPAO1 chromosome by PCR and cloned into the pCR2.1-1.8 vector at the TA cloning site downstream of the *lac* promoter to generate recombinant plasmid pLL1 (Table 1). The defect in biofilm formation by PW4830 was complemented by pLL1 (Figure 1D). These results suggest that *pvcA* is required for biofilm formation by MPAO1.

### The *pvc* Operon Contributes to Biofilm Formation by MPAO1

**Figure 2:**
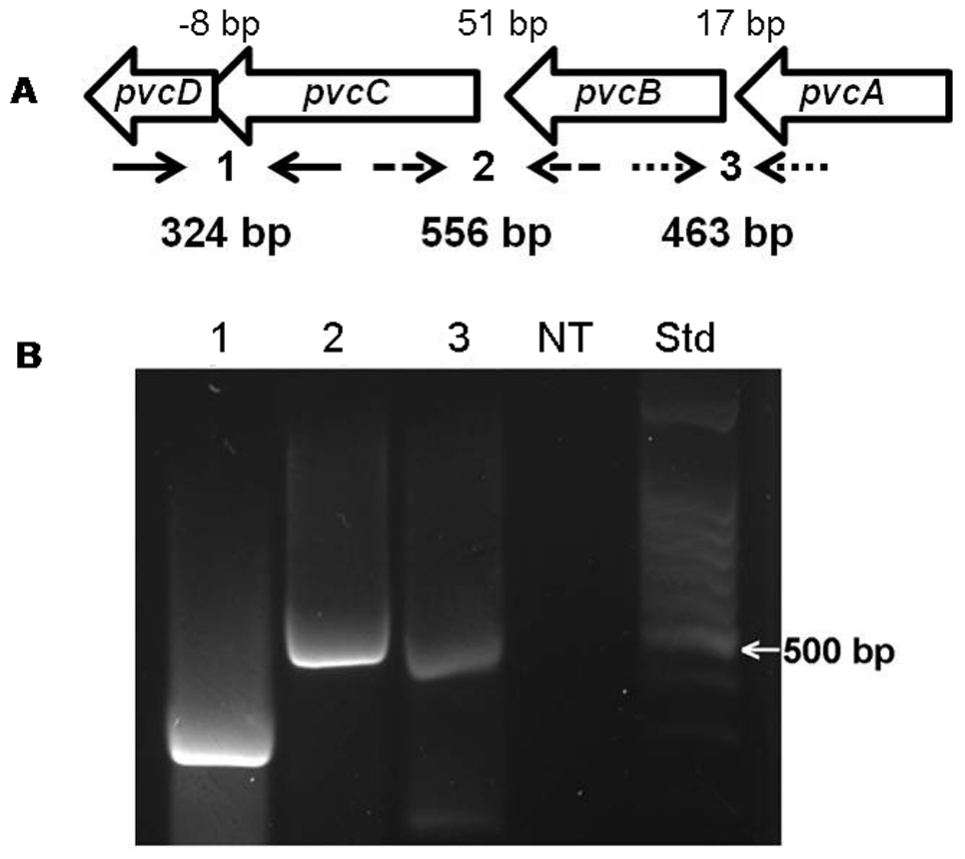
*pvcA-D* gene cluster constitutes an operon in MPAO1. Confirmation that the *pvcA-D* gene cluster constitutes an operon was accomplished through a series of RT-PCR experiments using primers that correspond to regions within adjacent genes. **A.** Order and direction of transcription of *pvcABCD* on the MPAO1 chromosome is indicated by block arrows. The intergenic region between each pair of genes is shown above and the location of the primers for each pair of genes is marked by the inward-facing arrows below: (1) *pvcC-pvcD,* solid; (2) *pvcB-pvcC*, dashed; (3) *pvcA-pvcB*, dotted. Sizes of the expected products are indicated below the primer locations. **B.** RNA from MPAO1 grown in LB broth for 16 h was obtained, processed, and reverse transcribed to produce cDNA as described in Materials and Methods. PCR reactions using each pair of primers were run and the products separated on a 1% (w/v) agarose gel and stained with ethidium bromide. (1) 324-bp product from *pvcC-pvcD*; (2) 556-bp product from *pvcB-pvcC*; (3) 463-bp product from *pvcA-pvcB*; (NT) no cDNA template control (Std) molecular size standard.

The contribution of *pvcA* to biofilm development in MPAO1 may also involve the function of the adjacent genes *pvcB*, *pvcC*, and *pvcD*. It has been suggested that the four genes constitute one operon [36]; yet, computer analysis suggested that the four genes constitute two operons: *pvcA-B*, and *pvcC-D* (data not shown) as the intergenic region between *pvcA-B*, *pvcB-C*, and *pvcC-D* are 17, 51, and −8 bp, respectively (Figure 2A). To determine which scenario is correct, we analyzed the transcript(s) produced from the four genes using RT-PCR and specific primer sets overlapping the intergenic regions between *pvcA* and *pvcB*, *pvcB* and *pvcC*, and *pvcC* and *pvcD* (Figure 2A). We detected three specific products from *pvcA-B* (463 bp), *pvcB-C* (556 bp), and *pvcC-D* (324 bp) (Figure 2B), indicating the presence of one transcript. Thus, *pvcABCD* constitute the *pvc* operon. Therefore, a mutation in any of the other genes besides *pvcA* should produce a phenotype similar to that observed in PW4830. Indeed, compared with MPAO1, PW4832, which carries a transposon insertion that inactivates *pvcB*, produced a significantly reduced biofilm (Table 1, Figure 1E, Figure S1). Additionally, this defect in biofilm formation was complemented by plasmid pLL2, which carries an intact copy of *pvcB* (Table 1, Figure 1F). Furthermore, the defects in biofilm formation in both PW4830 and PW4832 were complemented by plasmid pLL5, which carries the 3-kb region containing intact *pvcA* and *pvcB* (Table 1; data not shown). To analyze the role of the *pvcC-D* genes, we constructed an MPAO1 isogenic mutant (MPAO1Δ*pvcCD*) in which we deleted a 600-bp *BglII* fragment that encompasses portions of *pvcC* and *pvcD* (Table 1, Materials and Methods). Compared with MPAO1, MPAO1Δ*pvcCD* produced a significantly reduced biofilm (Figure 1G). These results suggest that the entire *pvc* operon contributes to the development of biofilm formation by *P. aeruginosa*. Further studies were conducted using strain PW4830.

### *pvc* does not Enhance Biofilm Formation by MPAO1 Through either Flagellum or Type IV Pili

**Figure 3:**
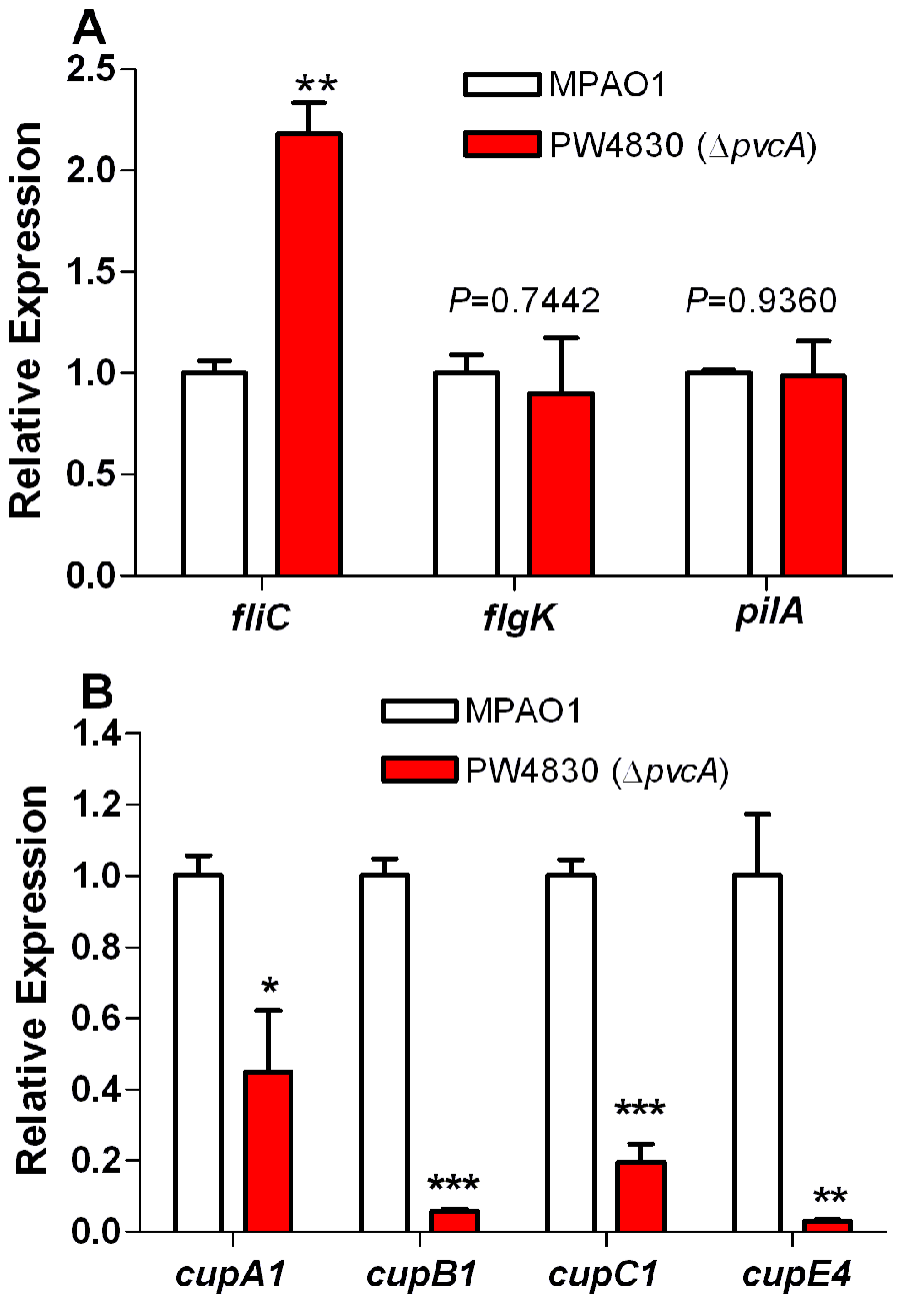
*pvcA* mutation does not interfere with the expression of *pilA* or *flgK.* *pvcA* mutation does not interfere with the expression of *pilA* or *flgK* but reduces *cup* gene expression. The relative level of expression for each gene in PW4830 (Δ*pvcA*) compared to MPAO1 was determined by RT-qPCR as described in Materials and Methods. The quantity of cDNA in different samples was normalized using 30S ribosomal RNA (*rplS*) as an internal standard. A. Mutation in *pvcA* does not interfere with the expression of either *pilA* or *flgK* but enhanced expressions of *fliC* in MPAO1. B. Mutation in *pvcA* affects expression of *cupA1, cupB1* and *cupC1* in MPAO1. Values in A and B represent the average of triplicate PCR experiments conducted on three independently obtained RNA preparations ± SEM (n = 3); *P*<0.05 (*); *P*<0.01 (**); *P*<0.001 (***).

Biofilm formation is a complex process involving three distinct stages: attachment, maturation, and dispersal [21]. Bacteria initiate biofilm by first reversibly and then irreversibly attaching to host tissues or abiotic surfaces, processes that depend on the flagellum and type IV pili [20]–[22]. Therefore, we next examined PW4830 for possible defects in the flagellum-mediated swimming motility or pili-mediated twitching motility. PW4830 showed comparable motility to MPAO1 on both swimming and twitching agar plates (data not shown). To confirm these results, we determined if the mutation in *pvcA* affected the expression of the flagellar and/or pilin genes. Using RT-qPCR, we analyzed the expression of the flagellar biosynthesis genes *fliC* and *flgK* [44] in MPAO1 and PW4830. Similarly, we examined the expression of the pilin biosynthesis gene *pilA* in both strains. The levels of expression of *flgK* and *pilA* in MPAO1 and PW4830 were comparable, while the level of *fliC* expression was higher, rather than lower (Figure 3A). These results suggest that *pvcA* does not enhance biofilm formation by MPAO1 through either the flagellum or the pili.

Other components that contribute to biofilm development in *P. aeruginosa* are the polysaccharides including Psl and Pel, which are required to maintain the biofilm structure in *P. aeruginosa* non-mucoid strains [45], [46]. Pel is required for pellicle development at the air-medium interface [47]. We examined the possibility that the *pvc* operon influences biofilm formation in MPAO1 through either Pel or Psl by analyzing the level of expression of *pelA*, *pslA*, and *pslD* in MPAO1 and PW4830. We detected no significant difference in the level of expression of these genes (Figure S2).

### *pvc* Influences Chaperone/Usher Pathway Systems in MPAO1

As the *pvc* operon does not affect biofilm formation in MPAO1 through either the flagellum or the type IV pili, *pvc* may affect it through the fimbriae. Fimbrial assembly in *P. aeruginosa* occurs through the proteins encoded by *cup* gene clusters, *cupA1-5, cupB1-6, cupC1-3*, and *cupE1-6* [26]. To examine the effect of *pvc* on the *cup* genes, we first compared the level of expression of the fimbrial subunit genes *cupA1*, *cupB1*and cupC1, between MPAO1 and PW4830 using RT-qPCR. The expression of *cupB1* and *cupC1,* in PW4830 was significantly reduced by 17- and five-fold, respectively, while the expression of *cupA1* was only reduced by 2.2-fold (Figure 3B). At this time, we decided to focus our efforts on analyzing the effect of *pvc* operon on the expression of *cupB* and *cupC* genes.

**Figure 4:**
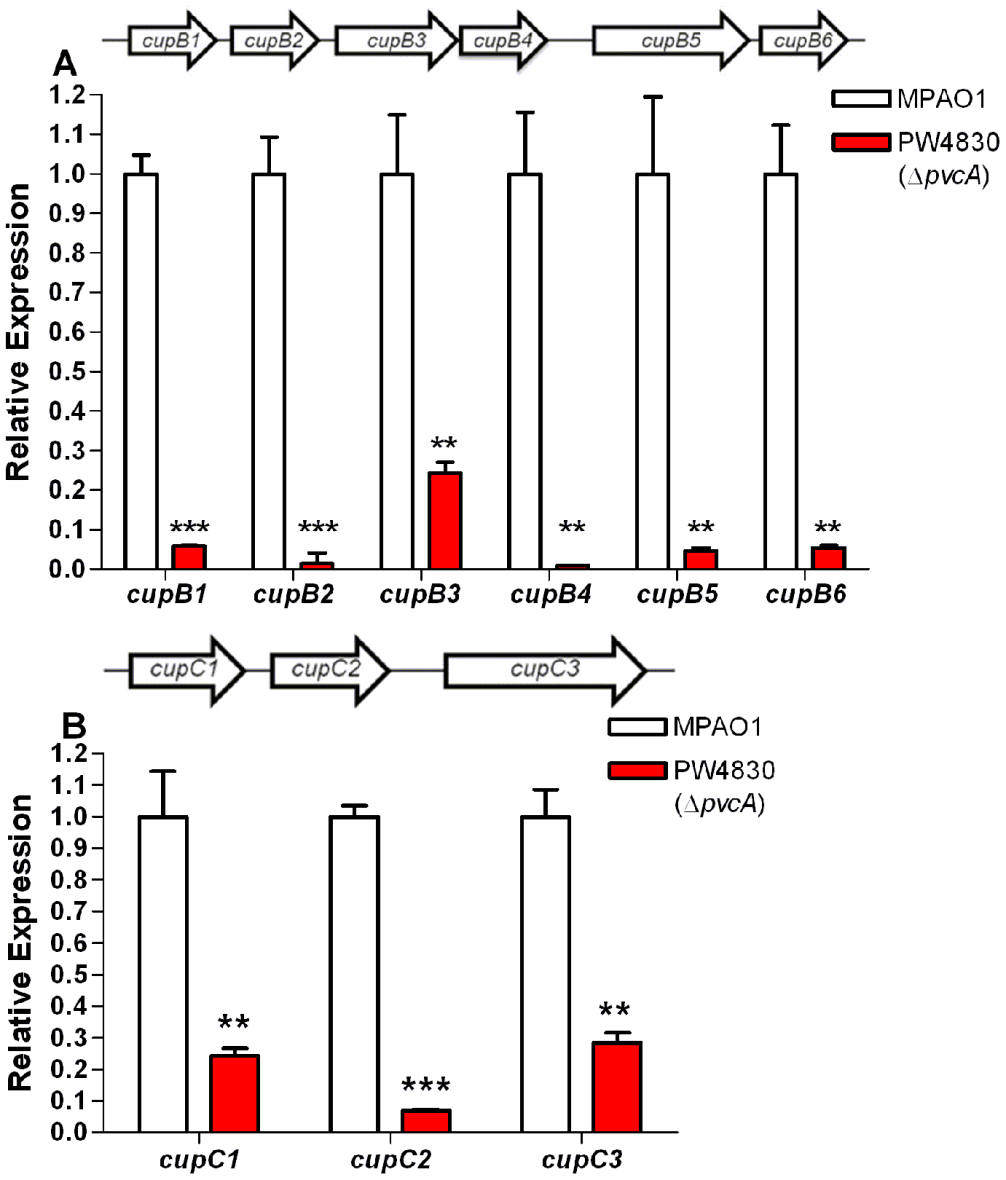
*pvcA* mutation reduces the expression of *cupB* and *cupC* gene clusters. The relative level of expression for each gene in PW4830 (Δ*pvcA*) compared to MPAO1 was determined by RT-qPCR. The quantity of cDNA in different samples was normalized using 30S ribosomal RNA (*rplS*) as an internal standard. **A.** *pvcA* mutation in MPAO1 significantly reduces the expression of all genes of the *cupB* gene cluster. Order and direction of transcription of *cupB1-6* on the MPAO1 chromosome is indicated by block arrows above the graph. **B.** *pvcA* mutation in MPAO1 significantly reduces the expression of all *cupC* genes. Order and direction of transcription of *cupC1-3* on the MPAO1 chromosome is indicated by block arrows above the graph. Values in A and B represent the average of triplicate PCR experiments conducted on three independently obtained RNA preparations ± SEM (n = 3); *P*<0.01 (**); *P*<0.001 (***).

Since *pvcA* affects the expression of the *cupB1* and *cupC1* fimbrial genes, we examined the effect of *pvcA* mutation on the expression of other genes within each cluster. In addition to the chaperones, fimbrial subunits, and the usher protein, the *cupB* cluster includes *cupB5*, which codes for a protein that is homologous to the *Bordetella pertussis* hemagglutinin and other nonfimbrial adhesion molecules [26], [48]. The level of expression of *cupB1-6* in PW4830 was significantly reduced by 17-, 64-, four-, 111-, 29- and 18-fold, respectively, compared with MPAO1 (Figure 4A). Similarly, the level of expression of *cupC1-3* in PW4830 was lower than MPAO1 by five-, 14-, and four-fold, respectively (Figure 4B). As we analyzed the effect of *pvc* on biofilm development using the same air-liquid assay that was previously used to analyze the role of the Cup pathways and the fimbriae in biofilm formation [24], [26], [28], these results suggest that the *pvc* operon influences biofilm formation in MPAO1 primarily through the fimbrial *cupB* and *cupC* gene systems. Results of preliminary experiments suggest that the *pvc* operon also regulates the expression of the *cupE1-6* genes as expression of *cupE4* was reduced in PW4380 (data not shown).

### *pvc* Enhances the Expression of *cupB* and *cupC* Through the *roc* Systems

**Figure 5:**
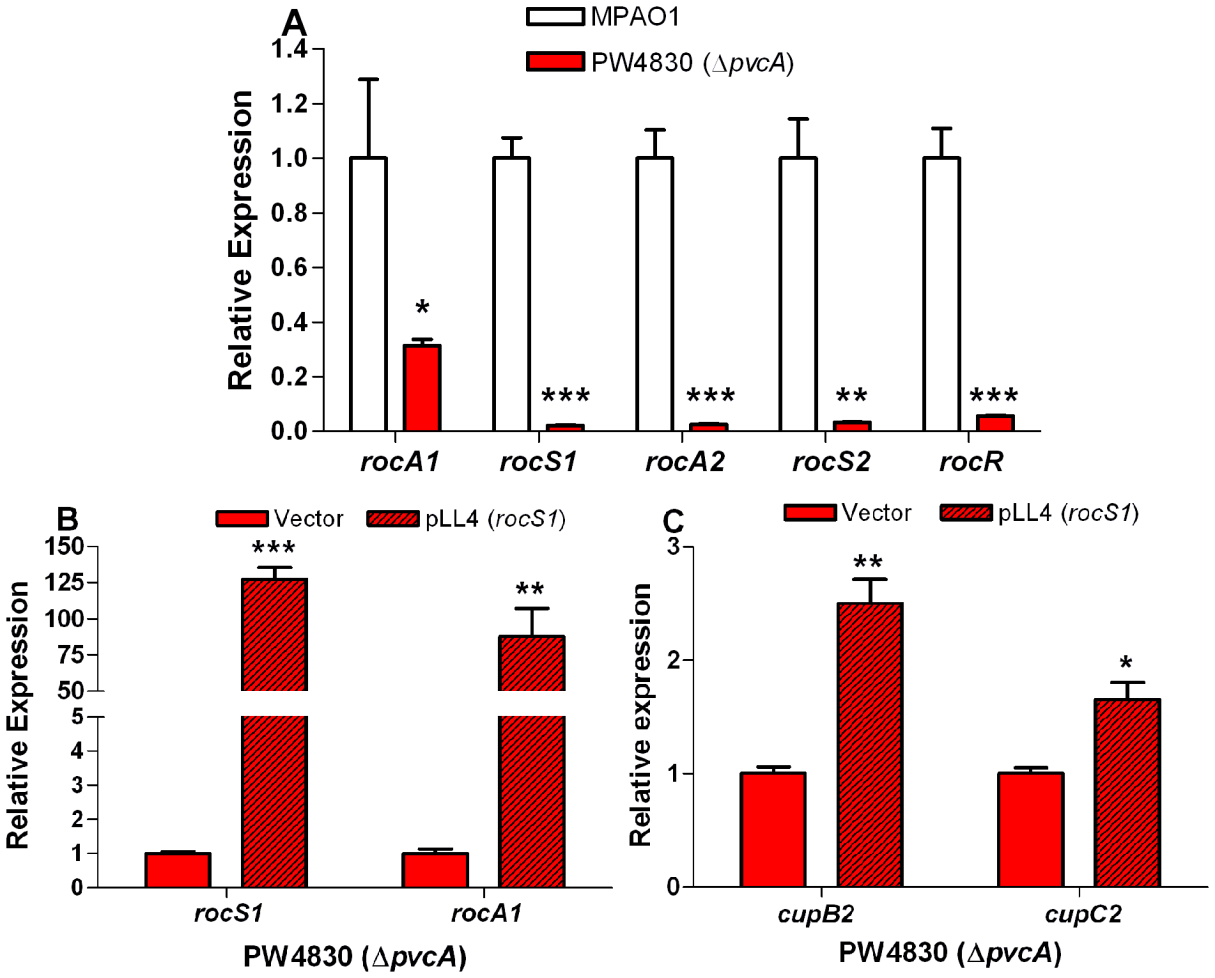
*pvcA* regulates *cup* genes through *roc1/roc2* systems. The relative level of expression for each gene in MPAO1 compared to PW4830 or PW4830/pLL4 compared to PW4830/pCR2.1-1.8 (vector control) was determined by RT-qPCR. The quantity of cDNA in different samples was normalized using 30S ribosomal RNA (*rplS*) as an internal standard. **A.** The *pvcA* mutation (PW4830) significantly reduces the expression of *rocS1*, *rocS2*, *rocA2*, and *rocR*, while expression of *rocA1* was less affected. **B.** Plasmid pLL4 carrying intact *rocS1* constitutively expressed from the *lac* promoter significantly increases the expression of *rocS1* and *rocA1* in PW4830 (Δ*pvcA*). PW4830 was transformed with pLL4 or pCR2.1-1.8 (vector control). **C.** Overexpression of *rocS1* from the *lac* promoter in pLL4 complements the defect of PW4830 in *cupB2* and *cupC2* expression. Values in A–C represent the average of triplicate PCR experiments conducted on three independently obtained RNA preparations ± SEM (n = 3); *P*<0.05 (*); *P*<0.01 (**); *P*<0.001 (***).

The expression of the *cupB* and *cupC* gene clusters is regulated by two different two-component systems, *roc1* and *roc2* [28]. The *roc1* system consists of RocS1, a membrane bound sensor kinase homologous to the *B. pertussis* multi-domain sensor kinase BvgS [30], [49]; RocA1, a conventional response regulator; and RocR, which contains an EAL domain and antagonizes RocA1 function [30]. The *roc2* system consists of an unorthodox sensor kinase, RocS2 and a conventional response regulator, RocA2 [30]. We examined the possibility that the *pvc* operon regulates the expression of one or more of these genes by comparing the level of their expression between MPAO1 and PW4830 (Δ*pvcA*). Compared with MPAO1, the levels of expression of *rocS1*, *rocR*, *rocS2*, and *rocA2* in PW4830 were reduced by 43-, 17-, 29-, and 39-fold, respectively; however, the level of *rocA1* expression was decreased by only three-fold (Figure 5A). Compared with other genes of the *roc1/roc2* systems, *rocA1* expression in MPAO1 is considerably lower (Figure S3). It is possible that in MPAO1, *rocA1* is not responsive to *rocS1* transcriptional enhancement. Yet, Kulasekara *et al.* [30] previously showed that *rocS1* overexpression from the *tac* promoter significantly increased *rocA1* expression in *P. aeruginosa* PAK. To confirm that *rocA1* responds to *rocS1*, we examined the effect of *rocS1* overexpression on the level of *rocA1* expression in MPAO1. Using PCR, we synthesized a 4060-bp fragment containing intact *rocS1* from MPAO1 chromosome and cloned it in pCR2.1-1.8 generating pLL4, in which *rocS1* is expressed from the *lac* promoter (Table 1), an *E. coli* promoter that is constitutively expressed in *P. aeruginosa* [50]. Compared with PW4830/pCR2.1-1.8, *rocS1* and *rocA1* expression was significantly enhanced in PW4830/pLL4 indicating the responsiveness of *rocA1* to *rocS1* in this strain (Figure 5B). As the *roc* systems regulate expression of the *cup* genes, we also examined whether pLL4 affects expression of *cupB2* and *cupC2* in PW4830. Overexpression of *rocS1* resulted in significant enhancement in expression of both genes (Figure 5C) suggesting that *pvc* regulates *cup* gene expression through the *roc* systems.

### *ptxR* Affects Expression of *cup* Genes Through Intact *pvc*

**Figure 6:**
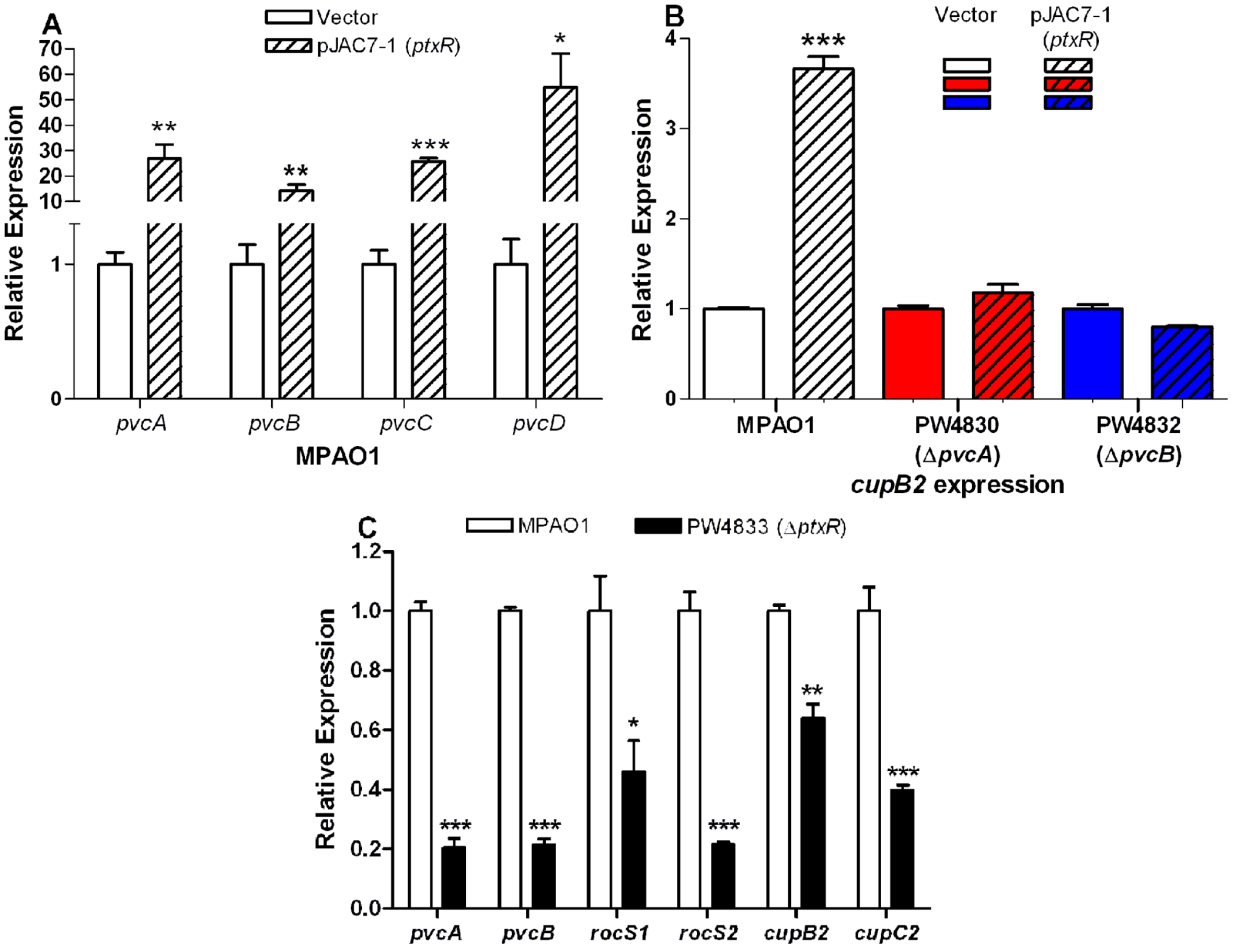
Regulation of the *cup* genes by *pvc* requires a functional ptxR. The relative level of expression for each gene in each pair of strains was determined by RT-qPCR. The quantity of cDNA in different samples was normalized using 30S ribosomal RNA (*rplS*) as an internal standard. **A. O**verexpression of *ptxR* from plasmid pJAC7-1 enhances expression of all genes in the *pvc* operon in MPAO1. MPAO1 was transformed with pJAC7-1 or p18.230 (vector control) and expression of *pvcABCD* in MPAO1/pJAC7-1 was compared with MPAO1/p18.230. **B.** Overexpression of the *pvc* operon by pJAC7-1 significantly enhanced *cupB2* expression in MPAO1 but not PW4830 (Δ*pvcA*) or PW4832 (Δ*pvcB*). Strains were transformed with pJAC7-1 or p18.230 (vector control) expression of *cupB2* in the strain carrying pJAC7-1 was compared to the strain carrying p18.230. **C.** Mutation in *ptxR* reduces expression of *pvc, roc,* and *cup* genes. Expression of *pvcA, pvcB, rocS1, rocS2, cupB2,* and *cupC2* were compared in PW4833 (Δ*ptxR*) and MPAO1. Values in A-C represent the average of triplicate PCR experiments conducted on three independently obtained RNA preparations ± SEM (n = 3); *P*<0.05 (*); *P*<0.01 (**); *P*<0.001 (***).

Stintzi *et al.* [36] previously provided evidence suggesting that *ptxR* positively regulates *pvc* expression in PAO1. Additionally, to analyze the different components encoded by the *pvc* operon in the *P. aeruginosa* strain PAK, Clarke-Pearson & Brady [37] overexpressed *pvc* using a *ptxR* plasmid. Therefore, we first examined the effect of *ptxR* on the expression of individual genes of the *pvc* operon in MPAO1. Either a vector control (p18.230) or pJAC7-1, which carries an intact copy of *ptxR* (Table 1), was introduced in MPAO1. The transformants were grown in LB broth for 16 h and the level of expression of different genes was determined. Compared with MPAO1/p18.230, the level of expression of *pvcA*, *pvcB*, *pvcC*, and *pvcD* in MPAO1/pJAC7-1 was increased by 26-, 14-, 25-, and 55-fold, respectively (Figure 6A). This confirms that a *ptxR* plasmid also drives overexpression of the individual genes of the *pvc* operon in MPAO1.

As *ptxR* regulates the expression of *pvc* in MPAO1, it would likely increase the expression of the *cup* genes, which are regulated by *pvc*. To test this possibility, we examined the expression of *cupB2* in MPAO1/p18.230 and MPAO1/pJAC7-1. The presence of pJAC7-1 increased *cupB2* expression in MPAO1 by 3.7-fold (Figure 6B). To confirm that *ptxR* enhances *cupB2* expression through *pvc*, we examined the level of *cupB2* expression in PW4830 and PW4832 carrying pJAC7-1 or p18.230. In contrast to MPAO1/pJAC7-1, the level of *cupB2* expression in PW4830/pJAC7-1 and PW4832/pJAC7-1 was not significantly different from that in the strains carrying p18.230 (Figure 6B). Similar results were obtained upon examination of the level of *cupC2* expression in MPAO1, PW4830, and PW4832 in the presence and absence of pJAC7-1 (data not shown).

Results described in Figure 6A and 6B were obtained using strains carrying multiple copies of *ptxR* (pJAC7-1 plasmid). To confirm that the phenomenon is truly *ptxR*-related and not artificially produced by multiple copies of *ptxR*, we compared the level of *pvcA*, *pvcB*, *cupB2*, and *cupC2* expression between MPAO1 and its *ptxR* mutant PW4833. We also compared the level of *rocS1* and *rocS2* in the two strains to further prove that *ptxR* regulates the expression of *cupB* and *cupC* through the *rocS* genes. The level of *pvcA*, *pvcB*, *rocS1*, *rocS2*, *cupB2* and *cupC2* expression in PW4833 was reduced by 5-, 4.7-, 1.6-, 2.5-, 2.2- and 4.6-fold respectively, compared to their expression in MPAO1 (Figure 6C).

### Paerucumarin Enhances the Expression of *cupB2*, *cupC2*, *rocS1* and *rocS2* in PW4830

**Figure 7:**
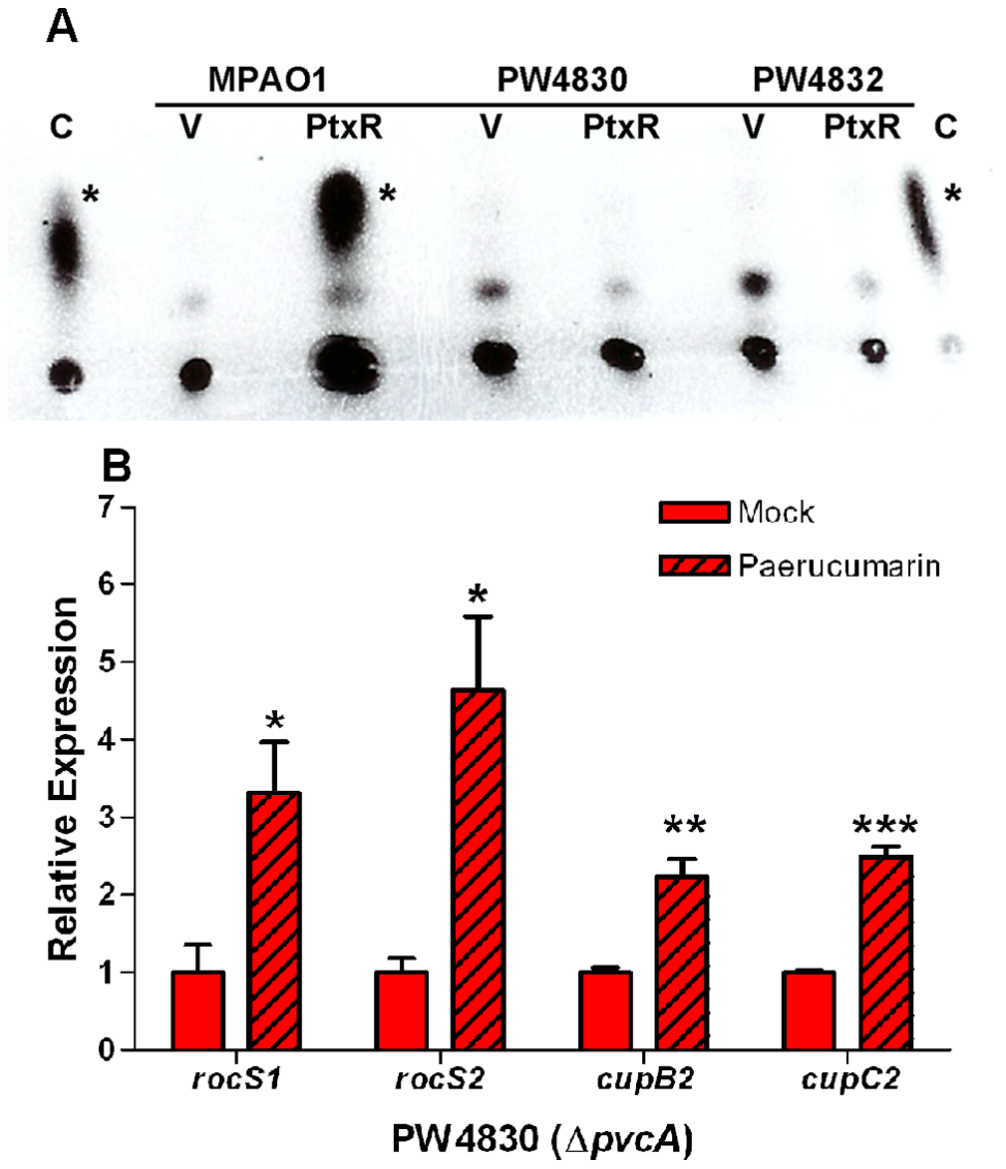
Exogenous paerucumarin enhances the expression of different genes within the *roc* and *cup* systems. The relative level of expression for each gene in each pair of strains was determined by RT-qPCR. The quantity of cDNA in different samples was normalized using 30S ribosomal RNA (*rplS*) as an internal standard. **A.** Over-expression of *pvc* by pJAC7-1 leads to accumulation of paerucumarin within the supernatant of MPAO1 but not those of PW4830 (Δ*pvcA*) and PW4832 (Δ*pvcB*). Strains carrying p18.230 (V) or pJAC7-1 (PtxR) were grown for 16 h at 37°C. Cells were pelleted and the supernatants isolated by centrifugation. Paerucumarin was extracted from the supernatant fractions by ethyl acetate and detected using TLC as described in Materials and Methods. Asterisk indicates position of paerucumarin. Purified paerucumarin served as a positive control (C). **B.** Exogenously added paerucumarin enhances the expression of *rocS1, rocS2*, *cupB2*, and *cupC2* in PW4830 (Δ*pvcA*). An overnight culture of PW4830 was subcultured into fresh LB broth to an OD_600_ of 0.02. A 15-µl aliquot of either ethyl acetate (mock) or purified paerucumarin 300 µM) was added at the time of subculturing. Cells were grown at 37°C for 16 h. The levels of gene expression in PW4830 plus paerucumarin were compared to the levels in mock-treated PW4830. Values in B represent the average of triplicate PCR experiments conducted on three independently obtained RNA preparations ± SEM (n = 3); *P*<0.05 (*); *P*<0.01 (**); *P*<0.001 (***).

Clarke-Pearson & Brady [37] previously showed that the major product of the *pvc* operon is a novel isonitrile-functionalized cumarin, paerucumarin. Paerucumarin was detected in the extract of the supernatant fraction of PAO1 in which *ptxR* was overexpressed [37]. Thus, we investigated the possibility that paerucumarin enhances the expression of *cupB2*, *cupC2*, *rocS1* and *rocS2* in MPAO1. We first confirmed that paerucumarin is produced by MPAO1/pJAC7-1 but not PW4830 or PW4832. Strains carrying either pJAC7-1 or p18.230 were grown in LB broth for 16 h at 37°C. The supernatant fraction was extracted with ethyl acetate and analyzed using thin layer chromatography. MPAO1/pJAC7-1 but not MPAO1/p18.230 produced a considerable amount of paerucumarin (Figure 7A). Despite the presence of the *ptxR* plasmid, neither PW4830 nor PW4832 produced detectable levels of paerucumarin (Figure 7A). Thus, production of paerucumarin requires functional *pvc*.

Next, we examined if exogenous paerucumarin affects the expression of *cupB2*, *cupC2*, and *rocS1* and *rocS2* in PW4830. Either ethyl acetate (mock) or purified paerucumarin was added to PW4830 and the expression of these genes was examined by RT-qPCR after 16 h of growth at 37°C. Compared with ethyl acetate, exogenously added paerucumarin significantly enhanced the expression of all four genes (Figure 7B). These results strongly suggest that exogenous paerucumarin bypasses the defect of PW4830 in the expression of the *cup* and *roc* genes. However, exogenously added paerucumarin had no effect on the expression of *pilA* and *flgK* genes (data not shown), which confirms the specificity of the paerucumarin effect on the expression of the *cup* genes. Besides the *cup* genes, the *roc1/roc2* regulon includes the *mexAB-oprM* operon, which is negatively regulated by the *roc2* system. Exogenously added paerucumarin significantly reduced the expression of *mexA* in PW4830 (data not shown), suggesting that paerucumarin may also regulate the expression of other genes of the *roc1/roc2* regulon. To confirm that the observed effect on the *cup* genes is unique to paerucumarin, we examined the effect of pseudoverdine on *cupB2* and *cupC2* expression. Pseudoverdine, the *N-*formyl adduct of paerucumarin, is also synthesized by the *pvc* operon; however, its function is unknown [37]. Therefore, we synthesized pseudoverdine using a commercial facility (Eburon Organics, Lubbock, TX). However, exogenously added pseudoverdine had no effect on either *cupB2* or *cupC2* expression in PW4830 (Figure S4, data not shown).

**Figure 8:**
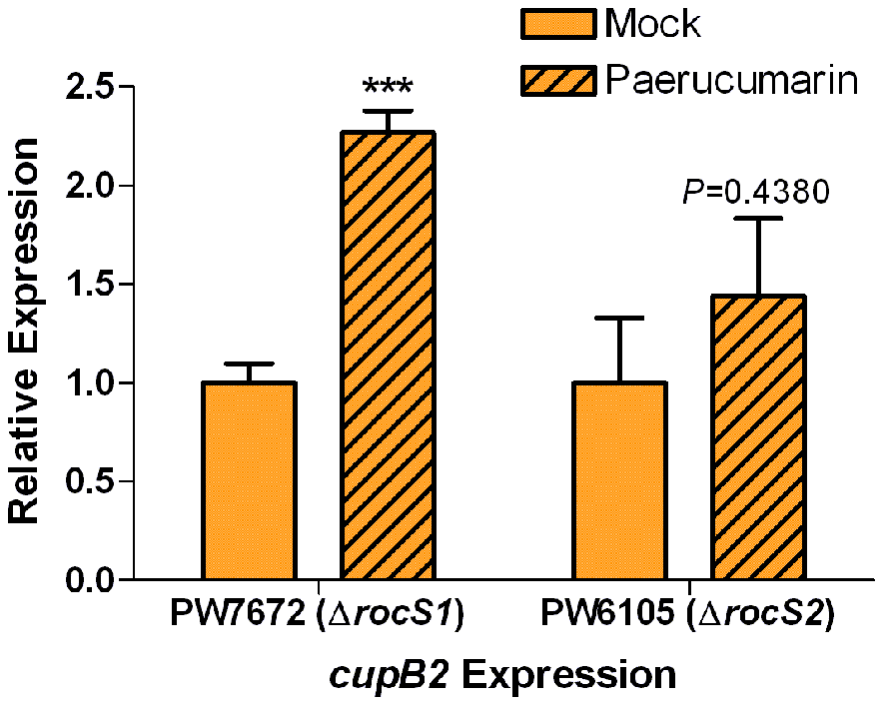
Exogenous paerucumarin enhances *cupB2* expression in the absence of *rocS1* and *rocS2*. Overnight cultures of PW7672 (Δ*rocS1*) and PW6105 (Δ*rocS2*) were subcultured into fresh LB broth to an OD_600_ of 0.02. A 15-µl aliquot of either ethyl acetate (mock) or purified paerucumarin (600 µM) was added at the time of subculturing. Cells were grown at 37°C for 16 h. The levels of *cupB2* gene expression were compared between mock treated and paerucumarin containing PW7672 and PW6105. The relative level of expression for *cupB2* in each pair of strains was determined by RT-qPCR. The quantity of cDNA in different samples was normalized using 30S ribosomal RNA (*rplS*) as an internal standard. Values represent the average of triplicate PCR experiments conducted on three independently obtained RNA preparations ± SEM (n = 3); *P*<0.001 (***).

To confirm that paerucumarin enhances the expression of *cup* genes through *rocS1*/*rocS2*, we determined the effect of exogenously added paerucumarin on *cupB2* expression in the *rocS1* mutant strain PW7672 and the *rocS2* mutant strain PW6105. At a concentration of 300 µM, paerucumarin had no effect on *cupB2* expression in either mutant (data not shown). However, at a concentration of 600 µM, paerucumarin significantly enhanced *cupB2* expression in the *rocS1* mutant (Figure 8). The same level of paerucumarin also slightly increased *cupB2* expression in the *rocS2* mutant, although this increase was not significant (Figure 8).

## Discussion

The results of this study indicate that the *pvcA-D* gene cluster constitutes an operon (*pvc*) (Figure 2). The entire *pvc* operon, rather than the effect of its individual genes, affects biofilm development in *P. aeruginosa* by regulating *cupB* and *cupC* fimbrial synthesis systems (Figures 1, 3 and 4). The four synthesis enzymes encoded by *pvc* are unlikely to be individually involved in fimbrial synthesis or biofilm development. Rather, our results strongly suggest that the effect is due to paerucumarin, the isonitrile-functionalized cumarin that is the product synthesized by the four PVC enzymes (Figure 7).

Initial studies indicated that *pvc* codes for pseudoverdine, a fluorescent bicyclic metabolite (cumarate derivative) that is structurally related to the chromophore of the pyoverdine molecule [35]. However, further studies identified the main product of the *pvc* operon as 2-isocyano-6,7-dihydroxycoumarin, or paerucumarin [37]. Paerucumarin, a novel molecule, is basically pseudoverdine in which the *N*-formyl group is replaced with an isonitrile moiety [37], [51]. PvcA belongs to the family of isonitrile synthases [51]–[53] and PvcB belongs to a family of alpha-keto glutarate dependent oxygenases [51], [54] that function in the production of isocyano derivatives of amino acids. Homologues for PvcA and PvcB exist in other bacteria including *Vibrio cholerae*, *Erwinia carotovora*, and *Legionella pneumophila* [37]. Similarly to *pvcA-D*, gene clusters that contain the PvcA/PvcB homologues in other bacteria synthesize metabolites with no known function [37]. Based on the results of this study, paerucumarin may function as an effector molecule that activates a *P. aeruginosa* transactivator. Upon its activation, the protein enhances the expression of several target genes including the *cupB* and *cupC* genes. In this capacity, paerucumarin would be similar to the *P. aeruginosa* QS-molecules including 3OC_12_-HSL, C_4_-HSL, and PQS that activate LasR, RhlR, and PqsR, respectively [55], [56]. Unlike QS-molecules, paerucumarin is not detected in the supernatant of *P. aeruginosa* under laboratory conditions; that is, in supernatants of cultures grown in LB broth at 37°C (Figure 7A) [37]. Paerucumarin was detected only in the supernatant of *P. aeruginosa* strains in which *pvc* is overexpressed either from a strong exogenous promoter or in the presence of a *ptxR* plasmid (Figure 7A) [37]. Despite this, wild-type strains of *P. aeruginosa* such as MPAO1 contain a sufficient amount of intracellular paerucumarin to influence bacterial functions including biofilm formation. Mutations within the *pvc* operon significantly reduced the expression of different *cup* gene clusters (Figures 3 and 4) as well as compromising biofilm development (Figure 1). Increasing paerucumarin production by overexpressing *pvc* through *ptxR* enhanced *cupB2* expression in MPAO1 but not in PW4830 (Δ*pvcA*) or PW4832 (Δ*pvcB*) (Figure 6B). Whether the environmental conditions within the host at certain infection sites induce *ptxR* expression leading to increased paerucumarin production is not known at this time.

**Figure 9:**
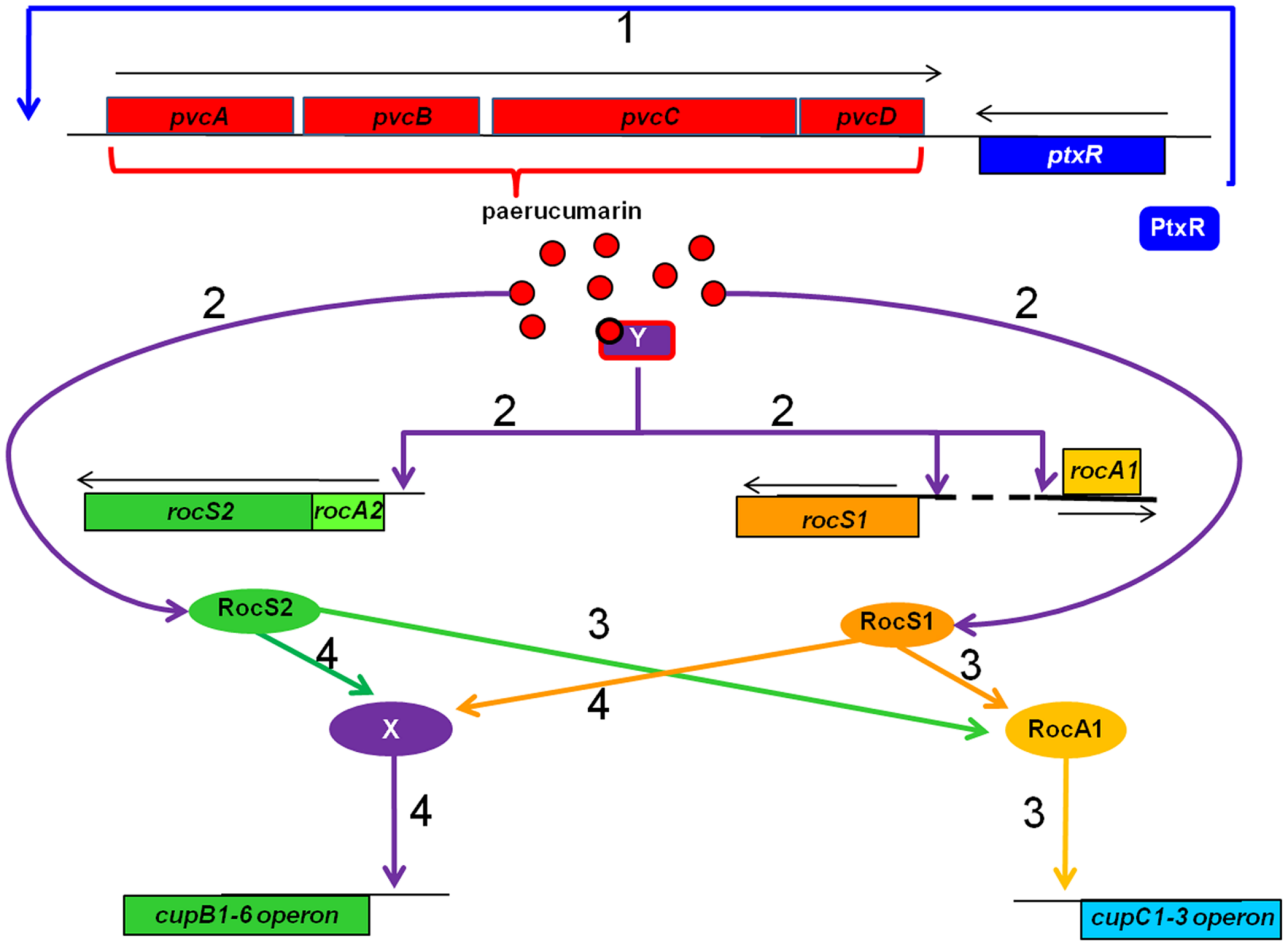
Diagram of the proposed regulatory circuits through which the *pvc* operon enhances the expression of *cup* genes . (1) PtxR enhances *pvc* expression and the proteins encoded by the *pvc* operon synthesize paerucumarin. (2) As a molecular signal, paerucumarin may activate the roc system through histidine kinases RocS1 and RocS2. Alternatively, paerucumarin may activate a potential transcriptional activator which enhances the expression of *rocS1-rocA1* operon, *rocS2,* and *rocA2*. (3) RocS2 and RocS1 then activate RocA1, which in turn enhances *cupC1-3* expression. (4) Both RocS2 and RocS1 enhance *cupB1-6* expression by activating an unknown regulator (X) [31]. Y indicates potential paerucumarin activated transcriptional regulator. Direction of expression is indicated by placement of genes above or below the black lines.

Based on these results, we propose the following model to explain our findings: PtxR enhances the expression of the *pvc* operon to produce more paerucumarin (Figure 9). Paerucumarin activates a potential transcriptional activator which enhances the expression of different *cup* genes (Figures 6 and 7) and facilitates biofilm development (Figures 1 and S1). At this time, we have no direct experimental evidence for such a transcriptional activator. However, extrapolating from the results of previous analyses, we propose PtxR may be this potential activator. In support of this possibility, we recently observed that paerucumarin exogenously added to the *ptxR* mutant PW4833 did not enhance the expression of either *cupB2* or *cupC2* (data not shown). However, further experiments will be conducted to fully explore the role of PtxR in regulating the *roc1/roc2* systems and the *cup* genes. PtxR belongs to the LysR family of transcriptional activators [39]. Most members of this family are activated by specific effector metabolites that bind to an effector binding or co-inducer binding domain within the carboxy terminus regions of these proteins [57]. Co-inducer binding induces a conformational change within the tertiary structure of the LysR protein which enhances its binding to the DNA target sequence [57]. For example, the co-inducers for CysB, NodD, and OxyR are *N*-acetylserine, flavonoids, and hydrogen peroxide, respectively [58]–[60]. Computer analysis indicated that the region spanning amino acid residues 99–297 within the carboxy terminus region of PtxR represents a potential co-inducer or effector binding domain (data not shown). However, that specific co-inducer has not yet been defined.

PtxR may resemble the *P. aeruginosa* PqsR/MvfR, another LysR DNA-binding protein that activates expression of the *pqsA-E* operon [61], [62]. PQS, the product of the *pqsA-E* operon, enhances PqsR binding to the *pqsA-E* upstream region thereby increasing *pqsA-E* expression and producing more PQS, which functions as a co-inducer [56]. Despite numerous attempts, including using multiple expression systems and commercial facilities, we have been unable to produce a sufficient amount of purified PtxR to examine its binding to the *pvc* upstream region, whether in the presence or absence of paerucumarin (data not shown). Currently, we are trying to determine the PtxR target sequence within the PAO1 chromosome using the ChIP-on-chip technique which does not require a purified protein [63].

The *pvc* operon and paerucumarin also positively regulate the expression of different *cup* genes (Figure 7). A *pvcA* mutation in MPAO1 significantly reduced the expression of the *cupB* and *cupC*, genes (Figure 3B). Additionally, exogenously added paerucumarin significantly enhanced *cupB2* and *cupC2* expression in the *pvcA* mutant strain PW4830 (Figure 7B). This effect is specific, as exogenously added pseudoverdine failed to enhance *cupB2* expression in the *pvcA* mutant (Figure S4). The expression of the *cupB* and *cupC* genes is controlled by the two well defined two-component regulatory systems *roc1* and *roc2* (Figure 9), which consist of a sensor kinase (RocS1 and RocS2) and a conventional response regulator (RocA1 and RocA2) [30], [31], [64]. The *roc1* system includes *rocR*, which antagonizes *rocA1* activity [30], [31], [64]. In response to environmental stimuli, *rocS1* activates *rocA1* which in turn enhances *cupC* expression; similarly, *rocS2* enhances the expression of *cupC* through *rocA1* but not *rocA2* [30], [31], [64]. Both *rocS1* and *rocS2* induce the expression of *cupB* through a mechanism that does not involve either *rocA1* or *rocA2* [30], [31], [64]. Compared with MPAO1, the expression of *rocA1*, *rocS1*, *rocA2*, *rocS2*, and *rocR* in PW4830 (Δ*pvcA*) was significantly reduced (Figure 5A). Exogenously added paerucumarin restored the expression of *rocS1* and *rocS2* in PW4830 (Figure 7B). Additionally, paerucumarin increased *cupB2* expression in the *rocS1* and *rocS2* mutants (Figure 8). However, such increase was significant only in the *rocS1* mutant (Figure 8). Since RocS1 and RocS2 are sensor kinases that respond to environmental stimuli, paerucumarin may represent the molecular signal to which RocS1 and RocS2 respond. Alternatively, paerucumarin might activate a potential regulator that enhances the expression of *cup* genes through *roc* systems (Figure 9).

We propose that the increased amount of paerucumarin enhances *rocS1* and *rocS2* expression (Figure 9). *rocS2* and roc*A2* are separated by only thirteen nucleotides on the *P. aeruginosa* genome and according to database of prokaryotic operons (DOOR) are computationally predicted to constitute an operon [65] (Figure 9). Therefore, *pvc* and paerucumarin may enhance the expression of both genes through a regulatory sequence within the upstream region of the operon (Figure 9). However, *pvc* and paerucumarin are likely to individually enhance *rocS1* and *rocA1* expression. RocS1 and RocS2 then activate RocA1, which enhances the expression of *cupC* genes (Figure 9). RocS1 and RocS2 also activate a potential, yet unidentified regulator which enhances *cupB* expression [30], [31], [64] (Figure 9). Therefore, RocS1 and RocS2 compensate for each other functionally and activate RocA1 as well as the unknown regulator in response to paerucumarin. As a result, exogenous paerucumarin was able to enhance *cupB2* expression in the *rocS1* mutant by enhancing the expression of the intact *rocS2* (Figures 8 and 9). A similar scenario occurs to a more limited extent in the *rocS2* mutant (Figure 8).

## Supporting Information

Figure S1 **Mutations in the pvc operon reduce biofilm formation in MPAO1.**
(TIF)

Figure S2 **Mutation in pvcA (PW4830) does not affect genes involved in polysaccharide biosynthesis.**
(TIF)

Figure S3 **The level of expression of rocA1 is low in MPAO1 compared to expression of the other roc genes.**
(TIF)

Figure S4 **Exogenous pseudoverdine does not complement the defect in cupB2 expression in the pvcA mutant (PW4830).**
(TIF)

Table S1 **Oligonucleotides used in this study.**
(PDF)

Text S1 **Crystal Violet Assay: supplemental methods and references for Figure S1.**
(PDF)
