# Supplementary material for: The pvc Operon Regulates the Expression of the Pseudomonas aeruginosa Fimbrial Chaperone/Usher Pathway (Cup) Genes
Source: PLoS One. 2013 Apr 30;8(4):e62735. doi: 10.1371/journal.pone.0062735 (PMC3639982; doi:10.1371/journal.pone.0062735)
Supplement: Table S1 — Oligonucleotides used in this study. (PDF) [file pone.0062735.s005.pdf]

Table S1. Oligonucleotides used in this study.

Gene / Locus ID	Name	Primer Sequence
**Cloning**		
pvcA, intact	*pvcA*_operon2R	GCCGTTGATCTGCACCA (paired with *pvcAB*_operon2F)
pvcB, intact	*pvcB*_operon2F	GACGAACAGGGCAACCAG (paired with *pvcAB*_operon2R)
pvcAB, intact	*pvcAB*_operon2F	GACAGTCTGAAGTTCGGCATC
	*pvcAB*_operon2R	ACGGCCACAGCATGACTT
rocS1, intact	*rocS1*F	AGGTACCAGAACCTTCGTCCAGAAGATGA
	*rocS1*R	AGAGCTCAACCGGAGTGTAGTCTTTTCC
**Analysis of *pvc* operon**		
pvcAB, intergenic	*pvcABF*	AAGTTCGGCATCCACATGA
	pvcABR	AGTAGCGGGTCAGGCTCTC
pvcBC, intergenic	*pvcBCF*	CAACCTGACCCTGTTGCAC
	pvcBCR	CCGGTAGTCGCCGTAGAA
pvcCD, intergenic	*pvcCDF*	CTACTGCGAGGCGATGGT
	pvcCDR	GCTGGGTCTTGAGGTCGTAG
**RT-qPCR**		
cgrA / PA2127	*cgrAF*	AAGAGCCTACCGCGTTCC
	cgrAR	GTCCAGGCGCATTGTTTC
cupA1 / PA2128	*cupA1F*	GCGGCAAACACTATCACATTC
	cupA1R	AACAGGGTGGTGAAATGCTC
cupB1 / PA4086	*cupB1F*	GTCAACTTCTCGGGCAACA
	cupB1R	ATGGAAACCTTCTTGTCATTGG
cupB2 / PA4085	*cupB2F*	CGCATCGCCTACCTGAA
	cupB2R	TTCCTGCTCCCCCTGAC
cupB3 / PA4084	*cupB3F*	TCCTCTGCCCGAAGGTTT
	cupB3R	TCCTGCCGTGGTTTTCC
cupB4 / PA4083	*cupB4F*	ACCCCGTCATCCGTCTC
	cupB4R	TTGTTCAGGTCTTCTTCCTTGTT
cupB5 / PA4082	*cupB5F*	CGACTATCCGCCCAACC
	cupB5R	CTTGAACAGGCAATCATCCA
cupB6 / PA4081	*cupB6F*	CCGATAATGGCGTGGATG
	cupB6R	ACCGCCGTCACCAGTTC
cupC1 / PA0992	*cupC1F*	AACCTGAACCTGGACTGTGG
	cupC1R	TTGCCTTCGCCATCTTTT
cupC2 / PA0993	*cupC2F*	AGGCTGATAATGGGGCAGA
	cupC2R	CGACGCAGATTTTTGTGGA
cupC3 / PA0994	*cupC3F*	TCGGCTGTGTATGGCTTGT
	cupC3R	TTTCGGTGGCGTTGATG
cupE4 / PA4651	*cupE4F*	CGTCTATCGCAACCAGCA
	cupE4R	CGTCGGGCACTTCGTC
fliC / PA1092	*fliCF*	TCCAACGCCTACGAGACC
	flicR	TCGCACCGTCCATCTTCT
flgK / PA1086	*flgKF*	GCGGCAAACACTATCACATTC
	flgKR	AACAGGGTGGTGAAATGCTC

**Gene / Locus ID**	Name	Primer Sequence
**RT-qPCR**		
lasB / PA3724	*lasBF*	GTTCTATCCGCTGGTGTCG
	lasBR	GCCCTTCTTGATGTCGTAGC
mexA / PA0425	*mexAF*	TCAACCTGCGCTACACCA
	mexAR	GCTACCGTCCTCCAGCTTC
oprM / PA0427	*oprMF*	GTTCGGGTTCCTGGTTGTT
	oprMR	TCGAGCAGGGTCAGGTAGTT
pelA / PA3064	*pelAF*	GTCGCAGAAACTCAAGGTCAA
	pelAR	CCTCCTCCAGCACATAGGG
pilA / PA4525	*pilAF*	AACCTGAACCTGGACTGTGG
	pilAR	TTGCCTTCGCCATCTTTT
pprB / PA4296	*pprBF*	CCGCCGACTACTACCAGAAA
	pprBR	GGCTGTTGCCGTGTACCT
pslA / PA2231	*pslAF*	GACGACGACCGCATCAC
	pslAR	CGCTGACCGCCTCCT
pslD / PA2232	*pslDF*	GCGTGCCCTGGAAGAAC
	pslDR	CTGGATGCGACCGATGA
pvcA / PA2254	*pvcAF*	CCTGTCGCTGTCGTTCCT
	pvcAR	CCTGGTAGGCGCTGATGT
pvcB / PA2255	*pvcBF*	ATTCCCCATCCTGCGTTT
	pvcBR	CGTGCAACAGGGTCAGGT
pvcC / PA2256	*pvcCF*	AAGGCGATCCACGAGATG
	pvcCR	CGAAGAACACAACAGCGAAG
pvcD / PA2257	*pvcDF*	CCTCTCGGCCCTGCTT
	pvcDR	TCGGGTAGCTGCGGTTC
rocS1 / PA3946	*rocS1F*	AGGCTGATAATGGGGCAGA
	rocS1R	CGACGCAGATTTTTGTGGA
rocR1 / PA3947	*rocR1F*	TCGGCTGTGTATGGCTTGT
	rocR1R	TTTCGGTGGCGTTGATG
rocA1 / PA3948	*rocA1F*	GTCAACTTCTCGGGCAACA
	rocA1R	ATGGAAACCTTCTTGTCATTGG
rocS2 / PA3044	*rocS2F*	CGCATCGCCTACCTGAA
	rocS2R	TTCCTGCTCCCCCTGAC
rocA2 / PA3045	*rocA2F*	TCCTCTGCCCGAAGGTTT
	rocA2R	TCCTGCCGTGGTTTTCC
