# Supplementary material for: The pvc Operon Regulates the Expression of the Pseudomonas aeruginosa Fimbrial Chaperone/Usher Pathway (Cup) Genes
Source: PLoS One. 2013 Apr 30;8(4):e62735. doi: 10.1371/journal.pone.0062735 (PMC3639982; doi:10.1371/journal.pone.0062735)
Supplement: Text S1 — Crystal Violet Assay: supplemental methods and references for Figure S1. (PDF) [file pone.0062735.s006.pdf]

Text S1: Supplementary Materials and Methods for Figure S1

Crystal Violet Assay for Biofilm Formation

The air-liquid interface method of biofilm development was used with some modification [1]. Overnight cultures of *P. aeruginosa* strains were subcultured at a starting OD_{600} of 0.02 into 3 ml of tryptone broth in polypropylene tubes (BD Biosciences) and incubated under static condition at 37°C for 18 h. Biofilms developed at the air-liquid interface were washed with sterile distilled water to remove planktonic cells. The crystal violet assay was conducted as previously described [2-4]. After thorough rinsing with sterile distilled water, the biofilms in the tubes were stained by the addition of 4 ml of 1 % (w/v) crystal violet. After incubation at room temperature for 30 min, the crystal violet solution was discarded. The tubes were then thoroughly washed with sterile distilled water and the stain eluted from the biofilms by the addition of 4 ml of 95 % ethanol. The eluted crystal violet was measured at 595 nm (A_{595}). Each experiment was repeated three times.

References

1. Kulasekara HD, Ventre I, Kulasekara BR, Lazdunski A, Filloux A, et al. (2005) A novel two-component system controls the expression of *Pseudomonas aeruginosa* fimbrial *cup* genes. Mol Microbiol 55: 368-380.
2. Déziel E, Gopalan S, Tampakaki AP, Lépine F, Padfield KE, et al. (2005) The contribution of MvfR to *Pseudomonas aeruginosa* pathogenesis and quorum sensing circuitry regulation: multiple quorum sensing-regulated genes are modulated without affecting *lasRI*, *rhlRI* or the production of N-acyl-L-homoserine lactones. Mol Microbiol 55: 998-1014.
3. O'Toole GA, Kolter R (1998) Flagellar and twitching motility are necessary for *Pseudomonas aeruginosa* biofilm development. Mol Microbiol 30: 295-304.
4. Schaber JA, Carty NL, McDonald NA, Graham ED, Cheluvappa R, et al. (2004) Analysis of quorum sensing-deficient clinical isolates of Pseudomonas aeruginosa. J Med Microbiol 53: 841-853.
